# Optimising fluid therapy during venoarterial extracorporeal membrane oxygenation: current evidence and future directions

**DOI:** 10.1186/s13613-025-01458-8

**Published:** 2025-03-19

**Authors:** Ali Jendoubi, Quentin de Roux, Solène Ribot, Victor Desauge, Tom Betbeder, Lucile Picard, Bijan Ghaleh, Renaud Tissier, Matthias Kohlhauer, Nicolas Mongardon

**Affiliations:** 1 Université Paris Est Créteil, INSERM, IMRB, Créteil, F-94010 France; 2 École Nationale Vétérinaire d’Alfort, IMRB, AfterROSC Network, Maisons-Alfort, F-94700 France; 3 Service d’anesthésie-réanimation et médecine péri-opératoire, DMU CARE, Assistance Publique- Hôpitaux de Paris (AP-HP), Hôpitaux Universitaires Henri Mondor, Créteil, 94010 France; 4 Faculté de Santé, Université Paris Est Créteil, Créteil, 94010 France; 5 Laboratoire de Pharmacologie, DMU Biologie-Pathologie, Assistance Publique des Hôpitaux de Paris (APHP), Hôpitaux Universitaires Henri Mondor, Créteil, 94010 France; 6 Department of Anesthesiology and Critical Care Medicine, Henri Mondor University Hospital, Assistance Publique - Hôpitaux de Paris (APHP), Inserm U955-IMRB, Équipe 03 “Pharmacologie et Technologies pour les Maladies Cardiovasculaires (PROTECT)”, École Nationale Vétérinaire d’Alfort (EnVA), Université Paris Est Créteil (UPEC), Maisons-Alfort, France

**Keywords:** Fluid management, Extracorporeal membrane oxygenation, Fluid responsiveness, Critically ill patients

## Abstract

Venoarterial extracorporeal membrane oxygenation (VA-ECMO) offers an immediate and effective mechanical cardio-circulatory support for critically ill patients with refractory cardiogenic shock or selected refractory cardiac arrest. As fluid therapy is routinely performed as a component of initial hemodynamic resuscitation of ECMO supported patients, this narrative review intends to summarize the rationale and the evidence on the fluid resuscitation strategy in terms of fluid type and dosing, the impact of fluid balance on outcomes and fluid responsiveness assessment in VA-ECMO patients. Several observational studies have shown a deleterious impact of positive fluid balance on survival and renal outcomes. With regard to the type of crystalloids, further studies are needed to evaluate the safety and efficacy of saline versus balanced solutions in terms of hemodynamic stability, renal outcomes and survival in VA-ECMO setting. The place and the impact of albumin replacement, as a second-line option, should be investigated. During VA-ECMO run, the fluid management approach could be divided into four phases: rescue or salvage, optimization, stabilization, and evacuation or de-escalation. Echocardiographic assessment of stroke volume changes following a fluid challenge or provocative tests is the most used tool in clinical practice to predict fluid responsiveness. This review underscores the need for high-quality evidence regarding the optimal fluid strategy and the choice of fluid type in ECMO supported patients. Pending specific data, fluid therapy needs to be personalized and guided by dynamic hemodynamic approach coupled to close monitoring of daily weight and fluid balance in order to provide adequate ECMO flow and tissue perfusion while avoiding harmful effects of fluid overload.

**Supplementary Information:**

The online version contains supplementary material available at 10.1186/s13613-025-01458-8.

## Background

Venoarterial extracorporeal membrane oxygenation (VA-ECMO) offers an immediate and effective option of a temporary mechanical circulatory support for critically ill patients with refractory cardiogenic shock. It is also increasingly used as a rescue strategy for refractory cardiac arrest [1, 2].

Refractory cardiogenic shock and post-cardiac arrest patients supported with VA-ECMO are characterized by high levels of cytokines and ischemia-reperfusion-related endothelial dysfunction leading to increased vascular permeability [3–5]. In addition, the exposure of circulating blood to the ECMO biomaterial surface could exacerbate the pro-inflammatory state [6]. All these mechanisms might contribute to vasoplegia and capillary leakage with subsequent intravascular fluid deficit requiring volume expansion and vasopressors to provide adequate blood flow and optimal tissue perfusion.

Fluid therapy is routinely performed as a component of initial hemodynamic resuscitation of ECMO supported patients but there are controversies regarding the optimal dose, type and endpoints of fluid resuscitation. In this narrative review, we will explore the rationale and the available evidence regarding fluid management in VA-ECMO setting. Vasopressor and inotrope management will not be addressed.

### The rationale for fluid therapy in ECMO setting

In vitro and in vivo experimental studies have shown that an intravascular volume deficit of 10% could lead to a reduction of ECMO blood flow by about 50% [7]. This ECMO low flow state can result in hypoperfusion and organ dysfunction. During the initial phase of ECMO, fluid resuscitation is often required to maintain adequate ECMO blood flow and to restore optimal global tissue perfusion.

ECMO blood flow is directly dependent on venous return (VR). As described by Guyton [8], VR is defined as the difference between mean systemic filling pressure (MSFP) and right atrial pressure (RAP) divided by resistance to venous return (RVR): VR = (MSFP - RAP) / RVR. The driving pressure for VR is the pressure gradient between MSFP and RAP, which then determines cardiac output.

The venous system can be divided into two compartments, the unstressed (70%) and the stressed (30%) volume. *The unstressed volume* (Vu), hemodynamically inactive, is the volume which fills the vasculature without a change in transmural pressure. However, this volume can be recruited by active venoconstriction and shifted to the stressed volume. *The stressed volume* (Vs) is the additional blood that generates positive transmural pressure via the elastic recoil of the vessel wall. An adequate fluid resuscitation exerts its therapeutic effect by increasing the stressed volume leading to an increase in MSFP and consequently in cardiac output [9–11].

ECMO blood flow depends on four factors: preload, afterload, pump speed and inflow cannula (position, length and diameter). The centrifugal blood pump is *preload dependent* with decreased flow in the event of profound hypovolemia or mechanical obstruction such as tamponade or tension pneumothorax (detailed below). The pump is also *afterload sensitive* with decreased flow in the event of high systemic afterload (increased mean arterial pressure MAP and systemic vascular resistances SVR). The determinants of venous return in the VA-ECMO setting are summarized in Supplementary Material (Figure S1).

Although Guyton’s concept offers a valuable framework to understand some features of ECMO flow - RAP relationship [12], there are still controversies about its applicability in VA-ECMO setting for several reasons: the interaction between extracorporeal flow and residual native cardiac output, the reduction of transpulmonary flow and the impact of venous collapse on flow restriction [13]. Taking into account these characteristics, venous return has a key role in ECMO flow, being the major determining factor of systemic perfusion especially in fully supported ECMO patients during the initial resuscitative phase. In addition to the recruitment of unstressed into stressed volume using vasopressors, fluid resuscitation remains the common choice to increase the maximum achievable ECMO flow [14].

## Intravascular volume deficit and triggers of fluid resuscitation in ECMO setting

**Fig. 1:**
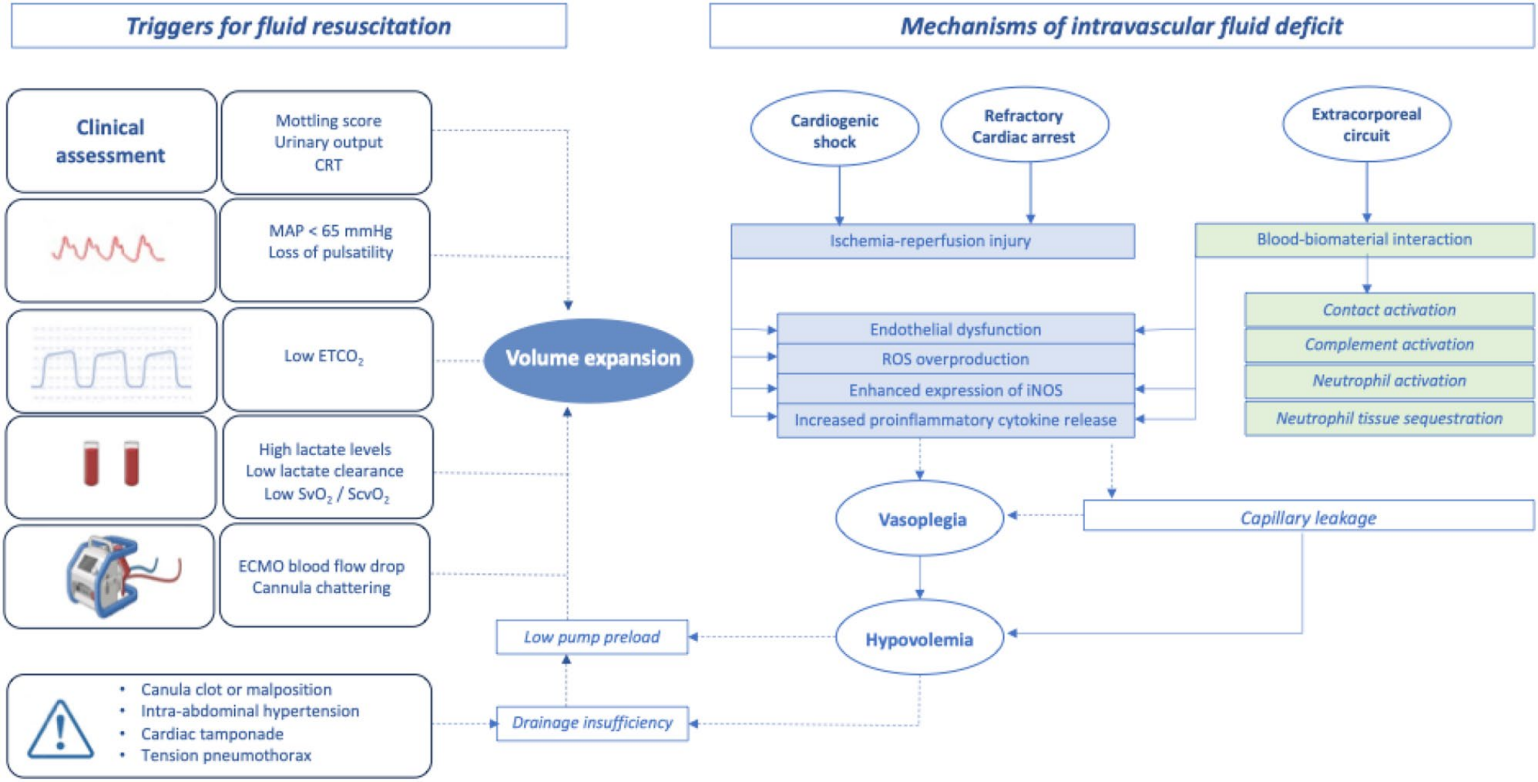
Intravascular volume deficit and triggers of fluid resuscitation in VA-ECMO setting. ROS: reactive oxygen species, iNOS: inducible nitric oxide synthase, ETCO_2_: End-tidal carbon dioxide, CRT: Capillary refill time, MAP: Mean arterial pressure, SvO_2_: Venous oxygen saturation, S_cv_O_2_: Central venous oxygen saturation

Intravascular volume deficit in ECMO setting can be attributed to three major factors: (i) native disease process, (ii) ECMO-related complications and (iii) potential mechanisms of drainage insufficiency which must be identified and addressed. The major mechanisms of hypovolemia and the triggers of fluid resuscitation are illustrated in Fig. 1.

### Native disease process

Cardiogenic shock patients exhibit a systemic inflammatory response to ischemia-reperfusion injury with endothelial dysfunction and subsequent capillary leakage. This proinflammatory state can lead to vasoplegia requiring vasopressors and large amounts of fluids to maintain an adequate intravascular volume and therefore an optimal blood flow [3–5].

### ECMO-related complications

The primary objective of VA-ECMO is to restore systemic hemodynamics and oxygen delivery. However, ECMO support is associated with potential complications. Among these complications, bleeding events and microvascular disturbances are of significant concern since they interfere with fluid management of VA-ECMO patients and impact negatively their outcome.

#### Bleeding and blood transfusion issues

Bleeding events occur in up to 30% of VA-ECMO supported patients [15] and are associated with hospital mortality [16]. As reported by an ELSO registry analysis [17], ECMO cannula and surgical site bleeding are the most frequent bleeding sites, followed by gastrointestinal bleeding, tamponade and hemorrhagic stroke [17]. There are many factors which contribute to the risk of bleeding in VA-ECMO setting including excessive heparin anticoagulation and hemostatic abnormalities such as hypofibrinogenemia, thrombocytopenia, platelet dysfunction, acquired von Willebrand syndrome and hyperfibrinolysis. The post-cardiotomy setting, ECMO duration more than 5 days and central cannulation site are also considered as risk factors of hemorrhagic complications [17]. As a result of these bleeding and coagulopathy risks, the transfusion requirements are high with reported red blood cell (RBC) transfusion rates of up to 90–100% of ECMO patients in some centers [18–19].

The optimal RBC transfusion threshold in VA-ECMO patients remains a matter of debate resulting in high practice heterogeneity among centers [19]. Because of the low level of evidence, Extracorporeal Life Support Organization (ELSO) guidelines were based only on expert opinion [20–21]. They recommended to target high transfusion thresholds by maintaining hematocrit above 40%, platelet count above 80 × 10^9^/L (100 × 10^9^/L in a bleeding patient) and normal fibrinogen levels between 2.5 and 3 g/L with infusion of fresh frozen plasma (FFP) or fibrinogen [21–22].

To date, a certain number of studies have investigated the impact of restrictive transfusion strategies on clinical outcomes [18]. A monocentric study has documented a significant reduction in RBC transfusion by 45% in postcardiotomy VA-ECMO patients after the implementation of a restrictive transfusion protocol [23]. In a recent systematic review and meta-analysis, the median transfusion threshold was 8 g/dL. A restrictive transfusion threshold of 8 g/dL was associated not only with a lower rate of transfusion but also with lower risks of mortality and AKI. These findings, although statistically significant, might be influenced by the poor methodologic quality and heterogeneity of the included studies [18].

The goal of RBC transfusion during ECMO is to increase blood oxygen delivery to tissues; however, it is associated with significant risk of complications such as overload, acute lung injury, increased infectious complications and alloimmunization. In addition, RBC transfusion may impair endothelial function and thereby reduce microcirculatory blood flow by different mechanisms: (i) increased blood viscosity, (ii) intravascular hemolysis with increased cell-free hemoglobin and compromised nitric oxide bioavailability and (iii) potential reduced oxygen release from stored RBCs [24–25].

Given concerns about transfusion-related complications as well as the emerging evidence supporting lower transfusion thresholds in other ICU populations including septic shock and cardiac surgical patients, adopting restrictive transfusion practices might be safer in the adult VA-ECMO population. Randomized controlled trials are needed to define the optimal transfusion strategy.

#### Microcirculatory dysfunction

ECMO support could have a negative impact on endothelial function for at least two reasons. First, it induces systemic inflammation, which may activate and damage the endothelium. Second, it generates non-pulsatile blood flow which may negatively impact the endothelial integrity. We will shed light on these two points.

##### The inflammatory response to ECMO

The blood-biomaterial interaction leads to simultaneous activation of contact and complement pathways [6]. The activation of factor XII-driven contact system triggers both intrinsic and extrinsic coagulation pathways leading to thrombin generation. The complement cascade system is also triggered upon ECMO initiation, predominantly via the alternate pathway with release of anaphylatoxins C3a and C5a and terminal complement complexes which induce early endothelial cell activation and promote the production of pro-inflammatory cytokines. These cytokines, notably TNF-α and IL-1β, trigger, in turn, the late activation of the endothelium with subsequent upregulation of adhesion molecules including P- and E-selectin that mediate recruitment, adhesion and trans-migration of activated neutrophils. The resulting tissue neutrophilic infiltration leads to ECMO-associated lung injury and end-organ damage [6]. Fluid resuscitation could potentially worsen organ injury by increasing tissue edema in this situation of endothelial barrier breakdown with vascular leakage.

##### Loss of pulsatility

Although ECMO support unloads the right ventricle and improves systemic blood flow, it is associated with increased left ventricle afterload due to the retrograde continuous flow. This may lead to a loss of pulsatility and left ventricle overload. The loss of pulsatile blood flow has been linked to endothelial dysfunction. When compared to non-pulsatile flow, pulsatile flow (i) enhanced the microcirculatory perfusion [26], (ii) alleviated inflammation response [27] (iii) preserved glycocalyx function by maintaining shear stress-mediated endothelial function [28] and (iv) protected endothelial integrity by up-regulating tight-junction proteins [28].

So, it is of paramount importance to restore or maintain the pulsatile activity of the native heart and aortic valve opening through therapeutic measures such as: fluid challenge in case of insufficient preload, ii) inotropic therapy and iii) left ventricle unloading by intra-aortic balloon pump (IABP) [29].

### ECMO drainage insufficiency

**Fig. 2:**
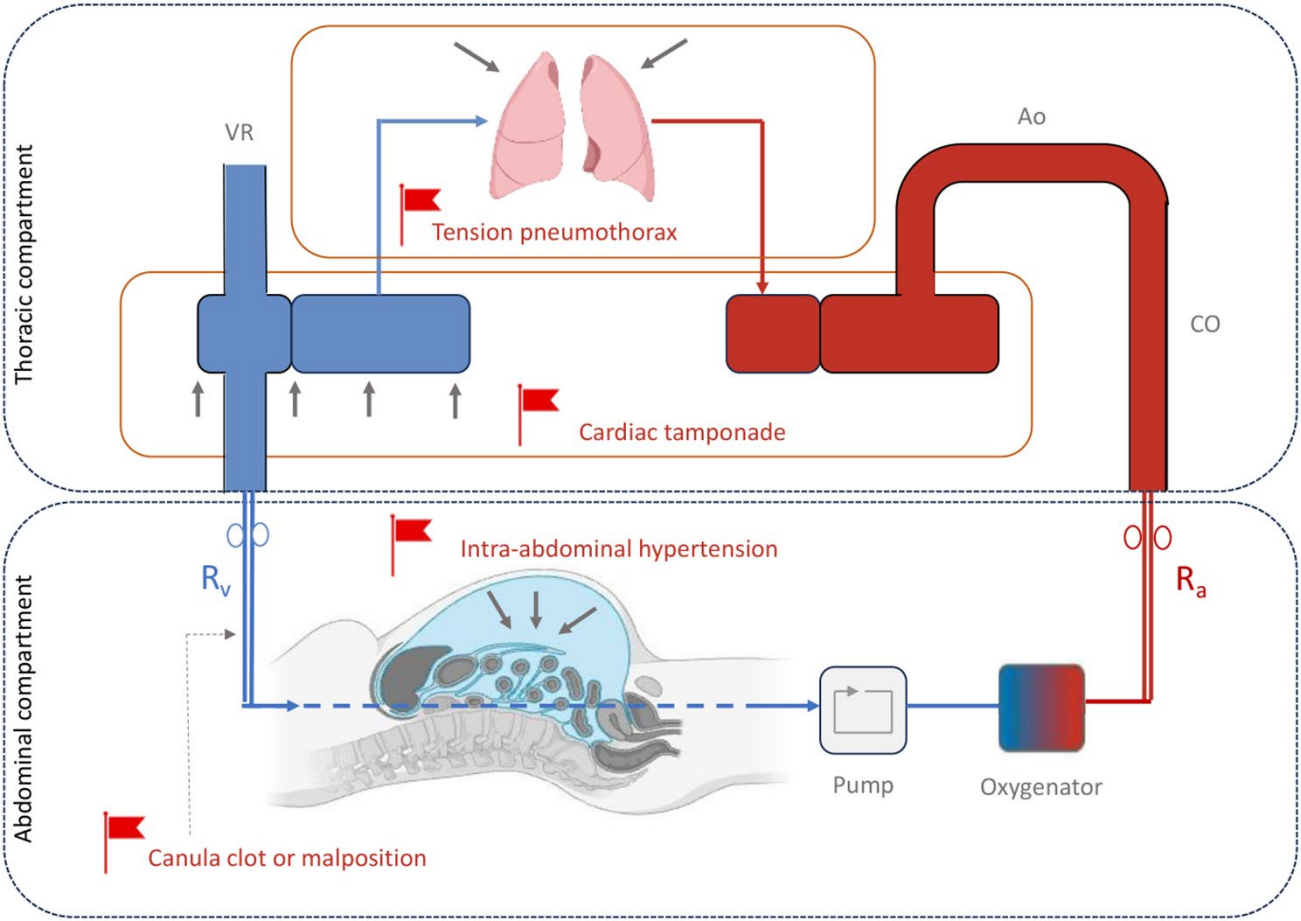
ECMO drainage insufficiency: potential causes and pitfalls. VR: venous return, R_v_: venous resistances, R_a_: arterial resistances, Ao: aorta, CO: cardiac output. Created with BioRender.com

In case of volume depletion or high pump speed, the excessive negative pressure generated by the pump may cause periodic venous collapse and subsequent decrease in ECMO blood flow. This can be visualized at the bedside as “circuit chugging or chattering” defined as intermittent suction events of the drainage cannula [30]. This common complication is often managed by fluid loading and/or by reducing pump speed. However, this issue requires an integrative and systematic approach in order to rule out other potential causes of impaired venous drainage such as canula clot or malposition, tension pneumothorax, cardiac tamponade or intra-abdominal hypertension. This thoughtful approach avoids reflexive fluid resuscitation [30] (Fig. 2).

In the context of severe volume depletion and drainage insufficiency, some caregivers use ECMO circuit as a vascular access port for fluid loading, but this practice exposes to over-resuscitation given the high fluid flow rates and risk of air embolism. Therefore, this practice should be avoided.

### Fluid resuscitation targets in VA-ECMO setting

**Fig. 3:**
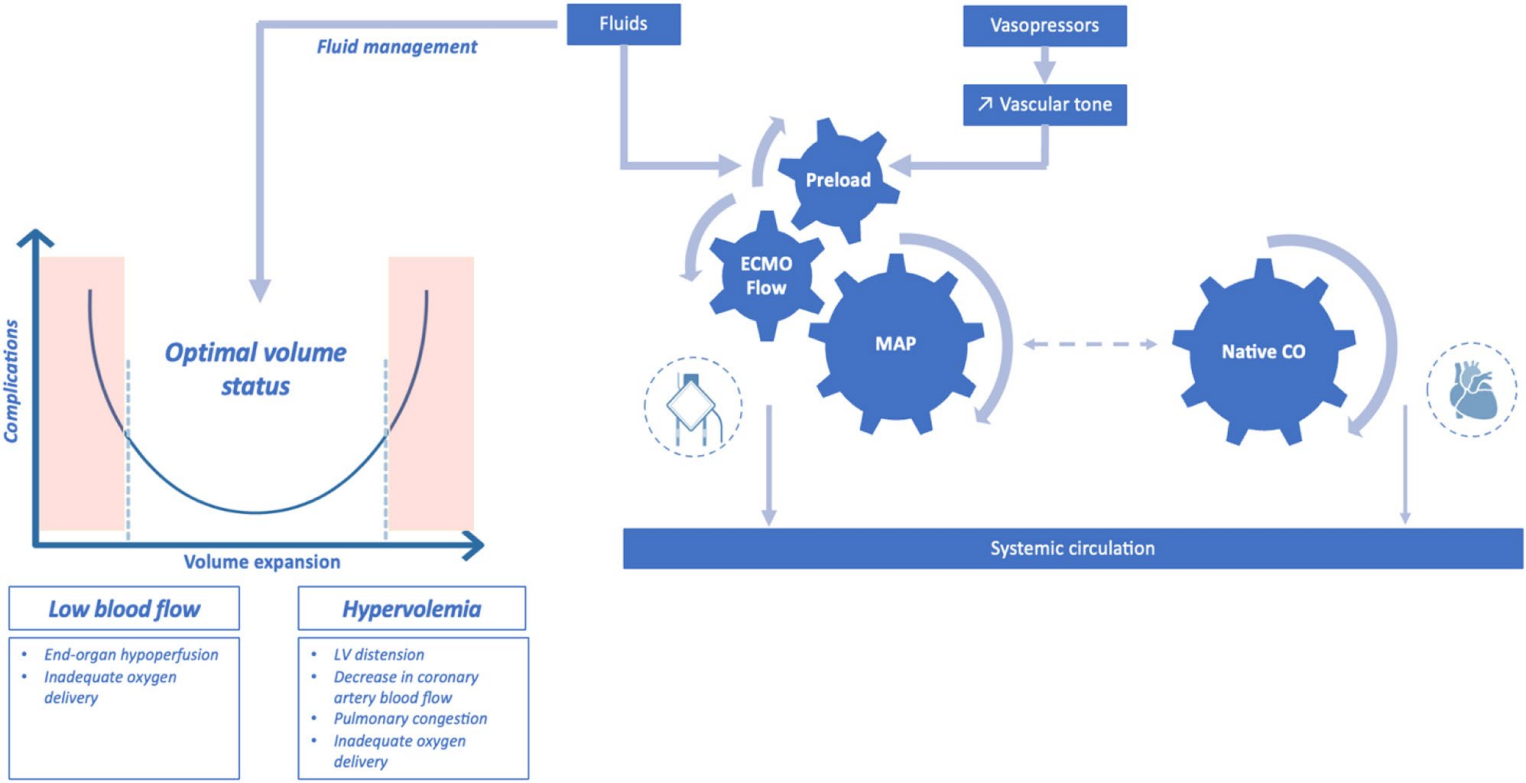
Key determinants of ECMO flow rate and complications associated with inadequate fluid management in veno-arterial ECMO supported patients. MAP, mean arterial pressure, CO: cardiac output

Arterial pressure optimization is crucial in the management of VA-ECMO patients. MAP depends on vascular tone and ECMO flow, which, in turn, depends on preload. In clinical practice, fluid resuscitation is the primary management option for caregivers to increase ECMO flow and blood pressure by increasing right ventricle preload (Fig. 3).

The optimal MAP target is not well established for VA-ECMO patients and remains challenging, balancing the risk of end-organ hypoperfusion at low levels of MAP with the risk of increased left ventricular afterload, wall stress and pulmonary edema when targeting higher MAP values.

These potential risks must be taken into account and require proactive and reactive management strategies aiming for three key factors: ECMO blood flow rate, vasopressors and fluids.

As for the MAP target, there is also a lack of evidence regarding the flow rate target. Current ELSO guidelines recommend titrating the flow rate in order to maintain an optimal tissue perfusion and adequate oxygen delivery [31–32]. In addition to ECMO flow, native cardiac output is also a major determinant of the systemic circulation, particularly during the recovery phase (Fig. 3).

All these factors need to be managed with a personalized dynamic approach combining (i) proactive measures such as goal-directed flow titration by monitoring of serum lactate level and markers of tissue perfusion and (ii) reactive measures such as fluid restriction or active fluid removal or even mechanical unloading strategies in case of left ventricular distension or pulmonary congestion.

### Dose and type of fluid therapy in VA-ECMO supported patients

Clinicians are often faced with questions regarding the fluid type choice and the optimal volume resuscitation strategy for ECMO patients.

### Type of fluid therapy in VA-ECMO setting

Crystalloids can be categorized based on their tonicity (isotonic, hypotonic, hypertonic), their electrolytic composition and their effects on acid-base status. According to Stewart approach, pH is regulated by three independent variables: partial pressure of carbon dioxide (pCO_2_), total amount of weak acids (A_TOT_) (mainly phosphate and albumin) and strong ion difference (SID), defined as the total difference between strong cations and anions mainly sodium and chloride [33].

Of note, the plasma SID, which is normally around 40 mEq/L, moves toward the SID of the infused crystalloids. Fluid-induced acid-base disturbances could be significant if large volumes are needed, as is the case during the initial resuscitative phase of VA-ECMO support.

In this context, for instance, the rapid infusion of large amounts of normal saline with a SID of zero, as an ECMO prime fluid or loading fluid, may result in dilutional hyperchloremic acidosis.

In order to manage this metabolic complication, balanced or buffered solutions have been postulated as alternatives to unbalanced normal saline. These solutions are characterized by lower chloride load and also the presence of buffers which are metabolized to bicarbonate producing an alkalizing effect such as lactate in lactated Ringer’s solution or gluconate and acetate in Plasmalyte [33]. The electrolytic composition, tonicity, pH, buffers and SID of commonly available crystalloids are summarized in Supplementary Material Table S1.

Despite the lack of data on the impact of the metabolic profile of crystalloids on clinical outcomes of VA-ECMO patients, some practice issues might be highlighted:

- The same fluid therapy, in terms of quantitative and qualitative composition, might have different metabolic effects and clinical outcomes depending on underlying conditions (early versus late ECMO implantation, organ failure status prior to ECMO initiation, the severity of pre-ECMO hypoperfusion and metabolic acidosis).
- It would be necessary to take into account the relative hypotonicity of some crystalloids such as lactate and acetate Ringer’s solutions. These solutions, slightly hypotonic about 270 mosmol/kg, are used as resuscitation fluids and mainly as maintenance solutions. In ECMO patients, maintenance and creep fluids represent an important part of daily inputs (15 to 23 mL/kg/d) [34] and, consequently, can lead to tissue edema in this context of capillary leak and endothelial permeability.
- The risk of coagulation impairment should be taken into account when managing fluids and blood products during ECMO support. Synthetic colloids and citrate-buffered crystalloids such as Optilyte^®^ should be avoided. Citrate metabolism could be severely impaired in ECMO patients with circulatory shock, hepatic failure and hypothermia [35].
- With regard to the choice of prime, ECMO circuit is frequently primed with an isotonic crystalloid solution [36]. Blood prime should be considered in patients weighting less than 20 kg or to compensate the blood lost during circuit exchange. Data on the choice of ECMO priming are scarce. There are some studies focusing on the use of balanced crystalloids for cardiopulmonary bypass (CPB) priming as alternatives to normal saline in order to prevent metabolic acidosis [37]. L-lactate, acetate and gluconate were among the buffers studied. Given the several distinctions between both techniques, it is necessary to assess the impact of these different crystalloid primes on the metabolic status and outcomes of ECMO patients [38, 39].

Among colloids, the role of albumin as a resuscitation fluid has been investigated in ECMO setting. In a retrospective registry study involving 283 extracorporeal cardiopulmonary resuscitation (ECPR) patients, the use of albumin was associated with improved survival compared to balanced crystalloids alone (43.9% vs. 27.6%, respectively after propensity score matching, *p* = 0.025) [40]. Another study reported a detrimental impact of hypoalbuminemia on the survival of ECMO supported cardiogenic shock patients. Indeed, it has been shown that pre-ECMO serum albumin level was an independent predictor of 30-day mortality (hazard ratio HR, 0.25; 95%CI 0.11–0.59, *p* = 0.002) even with higher amounts of albumin replacement [41]. In addition to these clinical findings, a recent animal study compared standard-care (norepinephrine + crystalloids) versus albumin resuscitation (albumin + standard-care) in a porcine model of ischemic refractory cardiac arrest resuscitated with VA-ECMO. Albumin infusion was highly effective in reducing crystalloid fluid loading within the first 6 h of ECMO support (1000 [1000–2278] ml vs. 17000 [10000–19000] mL, *p* < 0.001) but there was no significant difference between the two groups in terms of lactate clearance and sublingual capillary microvascular parameters [42]. Given the scarcity of data on this subject, specific studies should be performed to investigate the impact of albumin replacement in ECMO patients in terms of hemodynamics, microvascular endothelial function, tissue edema and organ damage.

### Dose of fluid therapy in VA-ECMO setting

Appropriate fluid management is pivotal during the inflammatory acute phase of VA-ECMO support. However, there is a lack of evidence regarding the most effective fluid resuscitation strategy (restrictive versus liberal approaches) and current ELSO guidelines provide no recommendation on this topic [43]. Two observational retrospective studies have shown that early liberal intravenous fluid therapy during the first 24 h adversely affects outcomes in adult VA-ECMO patients. The first one found that VA-ECMO patients with higher 3-hour fluid balance above the 75th percentile had a hazard ratio of death of 6.03 when compared to average survival with an area under the curve (AUC) of 0.726 [44]. In a second study involving 101 ECMO patients, the threshold of 38.8 mL/kg for the first 24 h of the ECMO run has been identified as predictive of mortality with a sensitivity of 60% and specificity of 83% AUC: 0.749 (95% confidence interval (CI): 0.653–0.843) [34].

Recent data from a large animal model have explored the impact of early fluid balance on renal function and organ edema in healthy pigs supported with VA-ECMO. Moderate versus extensive volume therapy strategies, based on the cumulative fluid administration, were compared during 10 h of ECMO run (3275 ± 263 mL vs. 5344 ± 834 mL respectively, *p* < 0.01). This showed impaired renal function and increased intestinal tissue edema in high-volume resuscitated group [45].

It is important to point out two key aspects related to the retrospective and monocentric design of the available studies. First, since no randomized controlled trial has ever addressed this issue, conclusions cannot be drawn regarding causality between fluid dosing and mortality. Indeed, the reported association between liberal fluid resuscitation and worse prognosis in ECMO patients should be viewed as a marker of their severity and their worse baseline status with increased capillary leakage leading to more suction events, more flow drops and more hypotensive episodes triggering fluid resuscitation. Second, if large amounts of fluids are associated with impaired outcomes, it does not mean that adopting a restrictive strategy is conversely associated with better outcomes.

### Fluid overload and outcomes

To note, it is of paramount importance to emphasize the ambiguity associated with the term “*fluid overload*”. This term should not be confused with the term “*hypervolemia*”. Hypervolemia is a state of intravascular overfilling and may be associated with edema. However, the presence of edema is not always associated with hypervolemia. Edema may occur with concomitant intravascular hypovolemia particularly in acutely ill patients with capillary leakage [46]. This distinction is challenging especially as the term “positive cumulative fluid balance” was used as a surrogate for fluid overload in several studies. Therefore, some authors suggest to avoid the misleading term “fluid overload” and to preferably use the term “fluid accumulation”. Fluid accumulation describes a continuum and no specific threshold of fluid balance and becomes clinically relevant once it leads to organ dysfunction called “fluid accumulation syndrome” [47].

There is growing evidence that fluid overload can negatively impact survival and organ function, particularly the kidney [48–57] (Table 1).

**Table 1:** Summary of studies describing the association of fluid overload with outcome in ECMO patients

Study	Design	Population	Fluid balance assessment	Outcomes
Staudacher et. 2017 [44]	Retrospective	VA-ECMO	FB H3, FB D1 and D3	↑ FB associated with ↑ mortality
He et al. 2018 [52]	Retrospective	VA-ECMO	FB D1, FB D3, FB D7	↑ FB associated with ↑ mortality
Besnier et al. 2020 [34]	Retrospective	VA-ECMO	FB D1, CFB (D1-D5)	↑ FB associated with ↑ mortality
Dong et al. 2023 [50]	Retrospective	VA-ECMO	CFB (D1-D4)	↑ FB associated with ↑ mortality
Taira et al. 2024 [51]	Retrospective	VA-ECMO	FB D1	↑ FB associated with ↑ mortality, unfavorable neurological outcome and AKI
Schmidt et al. 2014 [48]	Retrospective	Mixed (VA + VV)	CFB (D1-D3)	↑ FB associated with ↑ mortality
Kim et al. 2018 [49]	Retrospective	Mixed (VA + VV)	FB quartiles	↑ FB associated with ↑ mortality
Fong et al. 2020 [53]	Retrospective	Mixed (VA + VV)	CFB (D1-D3), (D1-D7)	↑ FB associated with ↑ mortality
Gunning et al. 2020 [54]	Retrospective	Mixed (VA + VV)	% FO > 10%	↑ % FO associated with ↑ mortality
Chiu et al. 2021 [55]	Retrospective	Mixed (VA + VV)	FB D1, CFB (D1-D3)	↑ FB associated with ↑ mortality
Lee et al. J 2021 [56]	Retrospective	Mixed (VA + VV)	CFB (D1-D3)	↑ FB associated with ↑ mortality
Thomas et al. 2022 [57]	Retrospective	Mixed (VA + VV)	FB D3	Negative FB on CRRT associated with ↓ mortality

**Abbreviations**: FB, fluid balance; CFB, cumulative fluid balance, %FO: percent fluid overload, D: day, CRRT: Continuous renal replacement therapy, AKI, acute kidney injury

#### Mortality

A positive fluid balance at ECMO day 3 was found to be an independent predictor of 90-day mortality in a mixed cohort of VA and VV-ECMO patients, even after adjusting for severity of illness and regardless of renal replacement therapy (RRT) use (odds ratio (OR): 4.02 [1.49–10.82] *p* = 0.006) [48]. Similar findings were reported by a larger cohort study [49], showing that ECMO patients with higher cumulative fluid balance during the first 3 days after ECMO initiation had a higher 90-day mortality (HR:1.76 [95%CI:1.37 to 2.27], *p* < 0.001) [49]. Two recent studies have focused on the subgroup of patients receiving ECPR and have demonstrated that higher fluid balance was consistently linked to poor outcomes. In the first study, excessive cumulative fluid balance during the first 4 days of the ECMO run was found to be independently associated with lower ICU survival (adjusted OR: 1.261, 95%CI: 1.091–1.375, *p* = 0.003) [50]. In the second study, cardiac arrest patients undergoing ECPR with the higher tertile of 24-hour fluid balance exhibited the highest odds ratio for mortality with a cutoff value of 5525 mL (OR: 1.97, 95%CI: 1.39–2.81, *p* < 0.001) [51].

#### Kidney outcomes

As with mortality, excessive positive fluid balance has been associated with poor renal outcomes. In a large mixed cohort of ECMO patients, higher fluid balance quartiles were associated with an increased acute kidney injury (AKI) incidence compared to lower quartiles (83.1% vs. 59.3%; *p* < 0.001 among patients with cardiac disease and 83.1% vs. 68.1%, *p* = 0.011 among patients without cardiac disease respectively) [49]. Another study demonstrated that positive fluid balance was significantly associated with AKI (OR, 1.04; 95%CI, 1.02–1.05; *p* < 0.001) and RRT use (OR, 1.05; 95%CI, 1.03–1.07; *p* < 0.001) in ECPR patients [51].

**With regard to neurological outcome**, excessive positive fluid balance in the first day following ICU admission was significantly associated with poor neurological outcome defined as cerebral performance category (CPC) score of 3 to 5 at discharge (OR, 1.03; 95% CI, 1.01–1.06; *p* = 0.005) in a cohort of OHCA patients receiving ECPR [51].

#### Lung injury

During VA-ECMO support, several factors might contribute to the increased risk of fluid overload-induced lung injury such as (i) native lung status before ECMO implantation with lung congestion in cardiogenic shock patients and post-cardiac arrest lung injury in ECPR patients [58]; (ii) ECMO-associated inflammatory response with lung injury and increased pulmonary vascular permeability and (iii) pulmonary congestion related to left ventricle pressure overload itself increased by retrograde flow. Data on the impact of fluid overload on lung function and recovery in VA-ECMO patients are very scarce. In a retrospective heterogeneous study, ECMO patients with positive fluid balance exhibited fewer mechanical ventilation free days at day 60 (median (IQR): 37 (0–48) days) compared to those with negative fluid balance (44 (16–54) days), *p* = 0.03 [48].

Pending more evidence, there are some measures aiming to reduce pulmonary congestion during VA-ECMO: (i) lung-protective ventilation; (ii) left ventricular unloading strategies in patients with increased left ventricle end-diastolic pressure, reduced pulsatility, persistent aortic valve closure and left ventricle stasis and (iii) reducing ECMO flow in patients with myocardial recovery and satisfactory peripheral perfusion.

### Hemodynamic monitoring and fluid responsiveness assessment

The purpose of the hemodynamic monitoring in ECMO-supported patients should integrate three dimensions: (i) native cardiac output, (ii) ECMO blood flow and (iii) assessment of the adequacy of systemic and regional perfusion [59]. Hemodynamic monitoring tools are useful for all phases of ECMO support from initiation (early inflammatory phase) to weaning (late recovery phase) in order to assess volume status and fluid responsiveness and to guide fluid removal. Prediction of fluid responsiveness helps to individualize fluid management and to avoid excessive volume resuscitation in VA-ECMO patients. Unfortunately, the accuracy of some dynamic preload indices such as stroke volume variation (SVV), pulse pressure variation (PPV), vena cava variations or transpulmonary thermodilution-derived volumetric variables might be affected by several factors: low tidal volume ventilation, inflow ECMO cannulas and indicator loss into the extracorporeal circuit [59–62]. Main monitoring devices and techniques with a summary of their applicability, validity and usefulness in predicting fluid responsiveness in VA-ECMO setting are summarized in Table 2.

**Table 2:** Main monitoring devices and techniques with a summary of their applicability, validity and usefulness in predicting fluid responsiveness in VA-ECMO setting

Hemodynamic monitoring techniques	Advantages and endpoints [with references]	Issues	Validity
**Prediction of fluid responsiveness**			
Dynamic preload indices SVV, PPV		Low tidal volume ventilation, arrhythmias, RV dysfunction, spontaneous breathing	Lack of validation studies
Pulse contour analysis (PCA)		Inaccurate CO measurements: in case of high vasopressor exposure and absent or low pulsatility	Lack of validation studies
Dynamic preload indices ΔSVC, ΔIVC		May be impacted by inflow ECMO cannulas	Lack of validation studies
Velocity time integral VTI_LVOT_ variation ΔVTI variation	VTI variation ΔVTI ≥ 10% after fluid challenge [59,60]	Intermittent measurement, operator dependent	Potentially valid
Passive leg raising		Could be impractical because of the immobilization of cannulated lower limbs	Further studies are needed
Trendelenburg maneuver	ΔVTI ≥ 10% induced by the Trendelenburg maneuver predicts FR [66]		Further studies are needed
Capillary Refill time variation ΔCRT	ΔCRT (-23%) after FC predicts a 10% increase of VTI [67]		Further studies are needed
**Native cardiac function**			
Focused echocardiography	Monitoring of LV unloading (LV size, aortic valve opening, LV stasis); Monitoring of LV recovery (EF, VTI)	Intermittent measurement, operator dependent	Potentially valid
Velocity time integral VTI_LVOT_	Assessment of native CO and response to fluids or inotropes	Intermittent measurement, operator dependent	Potentially valid
Transpulmonary thermodilution (TPTD)		Inaccurate CO measurements: unknown amount of the cold bolus is drawn into the ECMO circuit	Lack of validation studies
Pulmonary artery catheter PAC	PCWP a key parameter to assess LV unloading in VA ECMO supported patients Continuous monitoring of RAP, PAP and mixed SvO_2_	Thermodilution-based measurements of CO may be compromised by blood recirculating through the ECMO	Lack of validation studies
Pulse pressure PP	PP < 15 mmHg: predictive of low native CO [61]		Further studies are needed
PETCO_2_	EtCO_2_ < 14 mmHg: predictive of low native CO [61]		Further studies are needed
**Systemic and regional perfusion**			
NIRS (rSO_2_)	Cerebral NIRS: cerebral desaturations Limb NIRS: low rSO_2_ in case of ischemia of the cannulated limb [59]		Potentially valid
Capillary Refill time (CRT)	Useful and rapid bedside tool, could guide fluid resuscitation [59]		Potentially valid
Sublingual microcirculation (parameters: TVD, PVD, PPV%, MFI)	Assessment of microcirculatory disturbances in CS-patients supported with VA ECMO [62]	Expensive devices and time-consuming analysis	Potentially valid
SvO2 (pre-membrane lung oxygen saturation)	SvO_2_ of >70% generally reflects adequate ECLS support. [59]		Potentially valid
Low S_mv_O2 or S_cv_O_2_ values (< 65%)	Indicate a mismatch between oxygen delivery and consumption (trigger for Fluid resuscitation and/or RBC transfusion		Potentially valid
Elevated P_v-a_CO_2_ gap > 6 mm Hg	Could be used as a target of fluid resuscitation in ECMO patients [64,65]		Potentially valid

**Abbreviations**: FR: Fluid responsiveness, CO: cardiac output, LV: left ventricle, PCWP: Pulmonary capillary wedge pressure, RAP: right atrial pressure, PAP: pulmonary artery pressure), mixed SvO_2_: mixed venous oxygen saturation, EF: Ejection fraction, TVD: Total vessel density, PVD: perfused vessel density, MFI: Microcirculatory flow index, PPV%: percentage of perfused vessels, ΔSVC, ΔIVC: respiratory variations of superior (ΔSVC) and inferior vena cava (ΔIVC) diameters, NIRS: Near-infrared spectroscopy, rSO_2_: regional cerebral oxygen saturation, ETCO_2_: end-tidal carbon dioxide, S_mv_O2: Mixed venous oxygen saturation, S_cv_O_2_: central venous oxygen saturation, P_v-a_CO_2_ gap: Difference between the venous and arterial partial pressure of carbon dioxide

Drainage insufficiency, characterized by pre-pump chugging and flow drop, could be a sign of hypovolemia and might thereby trigger fluid loading. However, it requires a rational approach in order to rule out other causes such as: cannula misplacement or tamponade. A proposed bedside management algorithm of pre-pump chugging is outlined in Supplementary Material (Figure S2).

Among fluid response predictors in VA-ECMO setting, echocardiographic assessment of stroke volume changes (particularly changes in left ventricular outflow tract velocity time integral (VTI) following a fluid bolus challenge or provocative maneuver is the most used tool in clinical practice to predict fluid responsiveness [59, 60].

Of note, the pulsatility of the arterial waveform has a significant impact on the echocardiographic assessment of the native cardiac output. In fact, the loss of arterial pulsatility does not allow reliable measurement of the aortic VTI-derived cardiac output especially in fully supported ECMO patients. Echocardiography, while crucial in ECMO setting, has inherent limitations that impact its diagnostic accuracy such as low reproducibility in case of low native cardiac output and arrythmia; inability to provide bedside continuous monitoring and the need for expertise.

In this regard, it should be noted the importance of combining echocardiographic metrics with other hemodynamic data as a part of multiparametric approach and to understand the dynamic interaction between extracorporeal and native circulation. This latter point is particularly important given that VA-ECMO significantly decrease the transpulmonary blood flow generated by the native heart. Therefore, fluid loading could improve ECMO flow by increasing pump preload without a proportional improvement in native cardiac output.

Mixed venous oxygen saturation (S_mv_O2) reflects the adequacy between oxygen delivery (DO2) and the oxygen consumption (VO2) and it could be routinely assessed in VA-ECMO supported patients. However, it requires the placement of a pulmonary artery catheter [63]. Two surrogates could be used to assess the efficacy of tissue perfusion: (i) central venous oxygen saturation (S_cv_O2) from central venous catheter and (ii) pre-membrane lung oxygen saturation (S_pre_O2) estimated by blood gas analysis or saturation probe in the venous cannula. Low S_cv_O2 values below 65–70% indicate a mismatch between oxygen delivery and consumption. The aim is to improve oxygen delivery by increasing ECMO flow (via fluid resuscitation or increasing pump speed) and/or by RBC transfusion in order to increase oxygen carrying capacity.

In addition to SVO_2_, the gap between the venous and arterial partial pressure of carbon dioxide (P_v−a_CO_2_ gap) is also a marker of tissue hypoperfusion characterized by an increase in anaerobic CO_2_ production. It has been shown that an elevated P_v−a_CO_2_ gap > 6 mm Hg was associated with increased risk of mortality in cardiogenic shock patients supported by VA-ECMO. In addition to its prognostic value, this marker could be used as target of fluid resuscitation in ECMO patients if hemodynamic coherence is presumed preserved between macrocirculation and microcirculation [64, 65].

Two new useful bedside tools have been reported recently in VA-ECMO supported patients: the Trendelenburg maneuver [66] and the capillary refill time (CRT) [67]. It has been shown that an increase in VTI of at least 10% induced by the Trendelenburg maneuver was a reliable parameter in predicting fluid responsiveness with an AUC of 0.93 (95%CI: 0.81–0.98, 82% sensitivity and 88% specificity). However, ECMO patients with low pulse pressure (< 15 mmHg) were excluded, which limits generalizability of the results [66]. It has also been reported that a decrease in CRT by 23% after fluid challenge predict a 10% increase of VTI with a sensitivity of 92% [95%CI: 65–100] and a specificity of 73% [95%CI: 48–89]. In this study, CRT was measured using a standardized visual method [67]. The use of bedside automated capillary refill device might provide a reliable measure and could help to reduce interobserver variability [68].

Hemodynamic monitoring particularly dynamic approach allows individualization of fluid management. This individualized fluid strategy based on the optimization of stroke volume also called goal directed fluid therapy combines the potential benefits of fluids in terms of oxygen delivery and tissue perfusion and prevent the adverse effects of excessive fluid administration.

This strategy is currently recommended in major surgery to decrease the incidence of perioperative complications [69]. Scientific evidence is lacking in ECMO setting and there are no studies comparing optimized, restrictive and liberal fluid resuscitation strategies.

### Concept of fluid tolerance

There is an emerging paradigm shift to move from fluid responsiveness concept to fluid tolerance concept in critically ill patients [70]. This concept aims to prevent venous congestion and its deleterious impact on organ function. This venous congestion is quantified by Venous Excess Ultrasound (VExUS) Score assessing inferior vena cava and variations of hepatic, portal venous, and renal venous waveforms [71]. As there is a lack of data exploring this concept in VA-ECMO patients, it is unknown how far the VExUS method might be useful during the ECMO weaning phase for indicating and guiding fluid removal.

### Dynamic approach of fluid therapy in VA-ECMO setting

**Fig. 4:**
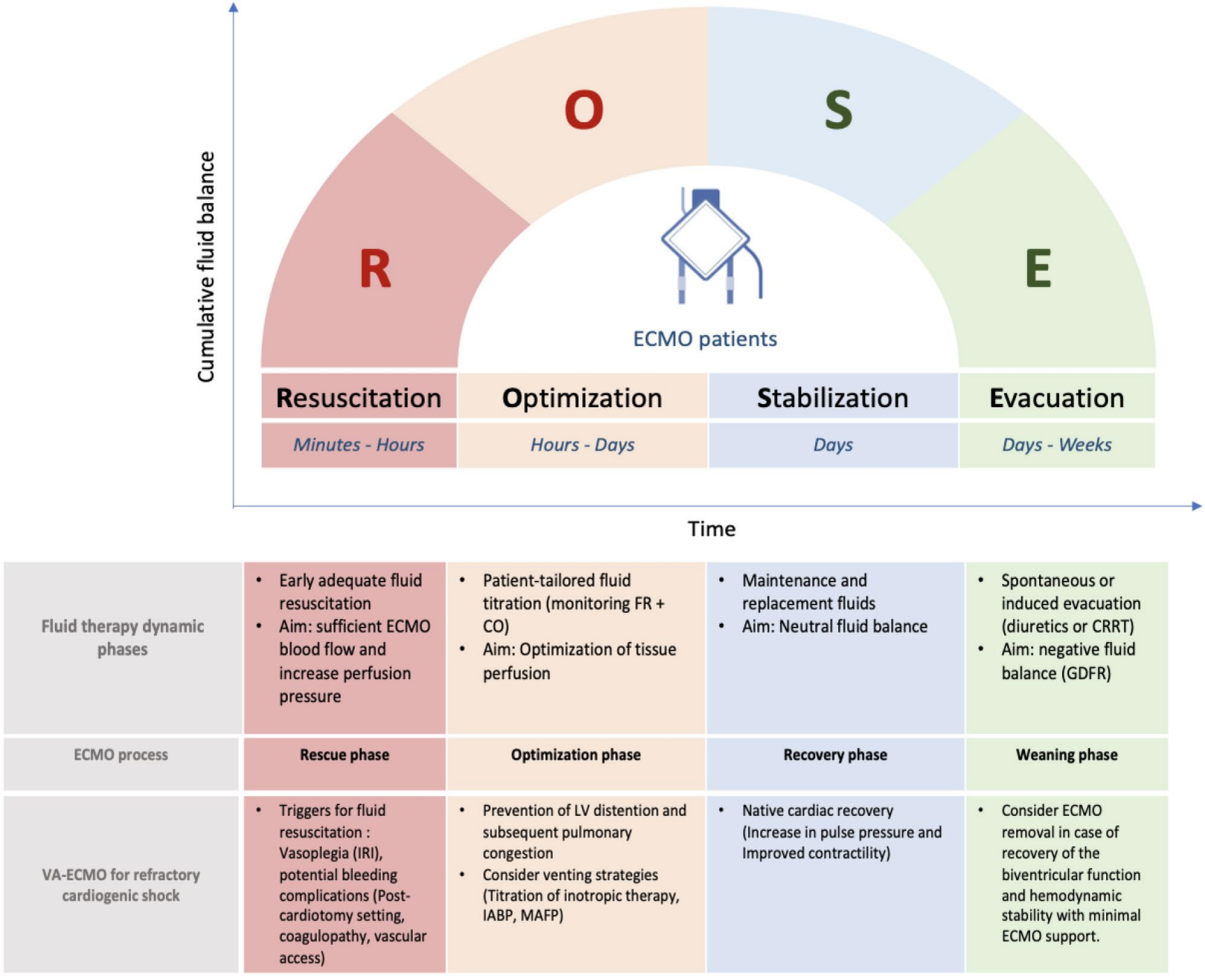
The ROSE concept of fluid therapy in VA-ECMO supported cardiogenic shock patients. IRI: ischemia-reperfusion injury, FR: Fluid responsiveness, CO: cardiac output, IABP: intra-aortic balloon pump, MAFP: Microaxial flow pump, MAP: mean arterial pressure, VTI: Velocity Time Integral, CRRT: continuous renal replacement therapy, GDFR: Goal directed fluid removal. *Adapted from Malbrain et al.* [73]

Four dynamic phases of fluid therapy for critically ill patients have been proposed and conceptualized through two acronyms S.O.S.D. or R.O.S.E. (Rescue or salvage, Optimization, Stabilization, Evacuation or de-escalation) [72, 73]. This conceptual model could also be adapted for VA-ECMO supported patients. Each phase of fluid therapy can be matched with the corresponding phase of ECMO support process, from initiation to weaning [74]. (Fig. 4)

The **Resuscitation phase** (R, phase 1) corresponds to the **rescue phase** of ECMO, occurring within the first hours after ECMO implantation. This acute phase is often associated with a profound intravascular fluid deficit due to capillary leakage and potential bleeding complications. This frequently requires large-volume fluid resuscitation to ensure sufficient ECMO blood flow.

The **Optimization phase** (O, phase 2) occurs within 24 to 48 h after ECMO initiation. The goal is to adjust tissue perfusion. In VA-ECMO-supported cardiogenic shock patients, the priority is to prevent and reduce left ventricular distention and subsequent pulmonary congestion by additional venting strategy using pharmacological (inotropic agents) or mechanical approaches (e.g., intra-aortic balloon pump or micro-axial flow pump).

The **Stabilization phase** (S, phase 3) evolves over days after shock resolution. During this homeostasis phase, fluid therapy is only used for ongoing maintenance and replacement fluids, targeting neutral fluid balance. This phase corresponds to the **recovery phase**. **N**ative cardiac recovery in VA ECMO patients is suggested by an increase in pulse pressure and by improved contractility on echocardiography.

Finally, the **Evacuation phase** (E, phase 4). This phase starts with spontaneous or induced evacuation (diuretics or RRT). It requires goal-directed fluid removal “de-resuscitation” in order to achieve negative fluid balance. This phase corresponds to the **ECMO weaning phase.**

The international ELSO guidelines recommend “to return the extracellular fluid volume to dry weight and maintain it there” [75]. Two points are worth noting: the first is about the choice between pharmacological and mechanical fluid removal modalities. The ELSO guidelines recommend diuretic treatment as an initial option. If the diuretic response is not sufficient to achieve negative fluid balance, RRT can be added, either through a dedicated catheter, or directly added to the ECMO circuit [76]. The second is about the optimal timing of RRT initiation. Although there are studies that have showed the beneficial effect of early initiation of RRT especially in pediatric patients supported with ECMO [77], the evidence supporting this practice in adults is lacking. A recent consensus concluded that there was no evidence of benefit for pre-emptive use of RRT in patients treated with ECMO [78]. Therefore, the decision to initiate RRT in ECMO patients should be based on usual indications for non-ECMO ICU patients.

### Knowledge gaps and future research directions

Currently, the available evidence is scarce and mostly consists of small-scale retrospective observational studies with heterogeneity of patients in terms of age, severity, ECMO type and indication. No prospective studies focusing on fluid management among VA-ECMO supported patients have been reported. Thus, several questions remain regarding notably the type (saline versus balanced crystalloids) and the dose (restrictive versus liberal) of fluid therapy.

In the light of available evidence, some practical implications could be drawn in order to optimize the fluid management of ECMO patients, avoid the deleterious effects of fluid overload and to limit blood product exposure, mainly (i) hemodynamic monitoring to achieve appropriate macrocirculatory and microcirculatory support, (ii) patient blood management with goal-directed transfusion strategy and (iii) define the place of second-line therapies such as vasopressin or albumin in order to decrease the amount of infused crystalloids during the initial resuscitative phase.

## Conclusions

Despite the paucity of evidence and limitations in the current data, this comprehensive review has enabled us to identify at least three areas for further research with potential practice recommendations: (1) fluid therapy should be personalized and guided by dynamic hemodynamic approach coupled to close monitoring of daily weight and fluid balance in order to provide adequate ECMO flow and tissue perfusion while avoiding harmful effects of fluid accumulation; (2) given the growing evidence of the negative impact of saline-induced hyperchloremia, a close monitoring of chloride levels is warranted among ECMO supported patients particularly during the early resuscitative phase. Albumin might be used as a second-line fluid therapy and (3) renal replacement therapy may mitigate fluid overload especially during VA-ECMO weaning phase.

## Electronic supplementary material

Below is the link to the electronic supplementary material.

Supplementary Material 1: Fig. S1 Determinants of venous return in VA-ECMO setting. Fig. S2 A proposed bedside management algorithm of pre-pump chugging. Table S1 The electrolytic composition, tonicity, pH, buffers and SID of commonly available crystalloids.

## Data Availability

Not applicable.
