# Supplementary material for: Optimising fluid therapy during venoarterial extracorporeal membrane oxygenation: current evidence and future directions
Source: Ann Intensive Care. 2025 Mar 19;15:32. doi: 10.1186/s13613-025-01458-8 (PMC11923310; doi:10.1186/s13613-025-01458-8)
Supplement: Supplementary file 1 — Supplementary Material 1: Fig. S1 Determinants of venous return in VA-ECMO setting. Fig. S2 A proposed bedside management algorithm of pre-pump chugging. Table S1 The electrolytic composition, tonicity, pH, buffers and SID of commonly available crystalloids. [file 13613_2025_1458_MOESM1_ESM.docx]

**Supplemental digital content**

| **ESM** | **Title** | **Page** |
| --- | --- | --- |
| **Figure S1** | **Determinants of venous return in VA-ECMO setting** | **2** |
| **Figure S2** | **Bedside management algorithm of pre-pump chugging in patients on venoarterial ECMO** | **3** |
| **Table S1** | **The electrolytic composition, tonicity, pH, buffers and SID of commonly available crystalloids** | **4** |

ESM: Electronic supplementary material

**Figure S1. Determinants of venous return in VA-ECMO setting.** The determinants of venous return are the MSFP, the RAP and the RVR according to the formula: venous return = (MSFP–RAP)/RVR. ECMO blood flow depends on four factors: preload (1. volemia status, 2. venous tone), inflow cannula (position, length and diameter) (3), pump speed (4) and afterload (5). MSFP: mean systemic filling pressure, RAP: Right atrial pressure, RVR: resistance to venous return, RA: right atrium, RV: right ventricle, LA: left atrium, LV: left ventricle, CO: cardiac output, MAP: mean atrial pressure, SVR: systemic vascular resistance, RPM: rotations per minute. Created with BioRender.com.

**Figure S2: Bedside management algorithm of pre-pump chugging in patients on venoarterial ECMO**

VA: veno-arterial, RPM: revolutions per minute, MAP: mean arterial pressure, SPO2 oxygen saturation, LPM: liters per minute, SVR: systemic vascular resistance, POCUS: point-of-care ultrasound

**Table S1. The electrolytic composition, tonicity, pH, buffers and SID of commonly available crystalloids**

|  | | **Unbalanced** | | **Balanced** | | | | | |
| --- | --- | --- | --- | --- | --- | --- | --- | --- | --- |
|  |  | **Isotonic** | **Hypertonic** | **Hypotonic** | | | **Isotonic** | | |
| **Composition** | **Plasma** | **NaCl 0.9%** | **NaCl 3%** | **Ringer's Lactate** | **Ringer's Acetate** | **Optilyte** | **Sterofundin ISO** | **Benelyte G1%** | **Plasmalyte 148** |
| **Tonicity** | | | | | | | | | |
| osmolality in vivo (mOsm/kg H_2_0) | 285-290 | 285 | 1026 | 256 | 270 | 273 | 286 | 278 | 271 |
| osmolarity in vitro  (mOsm/L) | 280-300 | 308 |  | 273 | 291 | 295 | 309 | 351 | 295 |
| **Strong ion difference** | | | | | | | | | |
| In-vivo SID (mEq/L) | 42 | **0** | 0 | **28** | 36.8 | 43 | **29** |  | **50** |
| **pH** | | | | | | | | | |
| pH | 7.35-7.45 | **4.5-7.0** | 5.5 | **6.0 - 7.5** | 6.0-8.0 | **5.5-7.5** | **5.1-5.9** | **5.5** | **7.4** |
| **Cationic and anionic composition** | | | | | | | | | |
| Na^+^ (mEq/L) | 136-145 | **154** | **513** | **130** | 137 | **141** | **145** | **140** | **140** |
| Cl^-^ (mEq/L) | 96-106 | 154 | 513 | **109** | **110** | **109** | **127** | **118** | **98** |
| K^+^ (mEq/L) | 4.5-5.0 | 0 | 0 | **4** | **4** | **5** | **4** | **4** | **5** |
| Ca^2+^ (mEq/L) | 2.2-2.6 | 0 | 0 | **3** | 1.65 | **2** | **2.5** | **1** | **0** |
| Mg^2+^ (mEq/L) | 0.8-1.0 | 0 | 0 | **0** | 1.25 | **1** | **1** | **1** | **3** |
| HCO_3_^-^(mmol/L) | 23-27 | 0 | 0 | 0 | 0 | 0 | 0 | 0 | 0 |
| **Buffers** | | | | | | | | | |
| Lactate (mmol/L) | < 2 | 0 | 0 | **28** | 0 | 0 | **0** | 0 | 0 |
| Acetate (mmol/L) | - | 0 | 0 | 0 | 36.8 | **34** | **24** | **30** | **27** |
| Malate (mmol/L) | - | 0 | 0 | 0 | 0 | 0 | **5** | 0 | 0 |
| Gluconate (mmol/L) | - | 0 | 0 | 0 | 0 | 0 | 0 | 0 | **23** |
| Citrate (mmol/L) | - | 0 | 0 | 0 | 0 | **3** | 0 | 0 | 0 |

Adapted from Langer et al. [33]
